# Emerging Trends and Innovations in the Treatment and Diagnosis of Atherosclerosis and Cardiovascular Disease: A Comprehensive Review towards Healthier Aging

**DOI:** 10.3390/pharmaceutics16081037

**Published:** 2024-08-03

**Authors:** Ibrahim Alradwan, Nojoud AL Fayez, Mohammad N. Alomary, Abdullah A. Alshehri, Alhassan H. Aodah, Fahad A. Almughem, Khulud A. Alsulami, Ahmad M. Aldossary, Abdullah O. Alawad, Yahya M. K. Tawfik, Essam A. Tawfik

**Affiliations:** 1 Advanced Diagnostics and Therapeutics Institute, Health Sector, King Abdulaziz City for Science and Technology (KACST), Riyadh 11442, Saudi Arabia; ialradwan@kacst.gov.sa (I.A.); nalfayez@kacst.gov.sa (N.A.F.); malomary@kacst.gov.sa (M.N.A.); abdualshehri@kacst.gov.sa (A.A.A.); aaodah@kacst.gov.sa (A.H.A.); falmughem@kacst.gov.sa (F.A.A.); kaalsulami@kacst.gov.sa (K.A.A.); 2 Wellness and Preventative Medicine Institute, Health Sector, King Abdulaziz City for Science and Technology (KACST), Riyadh 11442, Saudi Arabia; aaldossary@kacst.gov.sa; 3 Healthy Aging Research Institute, Health Sector, King Abdulaziz City for Science and Technology (KACST), Riyadh 11442, Saudi Arabia; alawad@kacst.gov.sa; 4 Department of Clinical Pharmacy, College of Pharmacy, King Saud University, Riyadh 11451, Saudi Arabia; ytawfik@ksu.edu.sa

**Keywords:** atherosclerosis, nanomedicine, gene therapy, immunotherapy, cell therapy, diagnostics

## Abstract

Cardiovascular diseases (CVDs) are classed as diseases of aging, which are associated with an increased prevalence of atherosclerotic lesion formation caused by such diseases and is considered as one of the leading causes of death globally, representing a severe health crisis affecting the heart and blood vessels. Atherosclerosis is described as a chronic condition that can lead to myocardial infarction, ischemic cardiomyopathy, stroke, and peripheral arterial disease and to date, most pharmacological therapies mainly aim to control risk factors in patients with cardiovascular disease. Advances in transformative therapies and imaging diagnostics agents could shape the clinical applications of such approaches, including nanomedicine, biomaterials, immunotherapy, cell therapy, and gene therapy, which are emerging and likely to significantly impact CVD management in the coming decade. This review summarizes the current anti-atherosclerotic therapies’ major milestones, strengths, and limitations. It provides an overview of the recent discoveries and emerging technologies in nanomedicine, cell therapy, and gene and immune therapeutics that can revolutionize CVD clinical practice by steering it toward precision medicine. CVD-related clinical trials and promising pre-clinical strategies that would significantly impact patients with CVD are discussed. Here, we review these recent advances, highlighting key clinical opportunities in the rapidly emerging field of CVD medicine.

## 1. Introduction

Cardiovascular diseases (CVDs) have become the leading causes of morbidity and mortality globally, causing the death of approximately 18 million annually. This is predicted to reach 23.6 million lives annually by 2030, primarily due to heart attacks and strokes that account for 85% of the cases [1]. The primary causes of cardiovascular dysfunction include behavioral risk factors such as tobacco use, unhealthy diet and obesity, physical inactivity, and harmful use of alcohol [2]. These behavioral factors can result in the accumulation of lipids and fibrous elements within the arterial wall due to the disturbance of cholesterol and immune system equilibrium, resulting in atherosclerotic plaque formation in the walls of the vasculature system [1].

**Figure 1:**
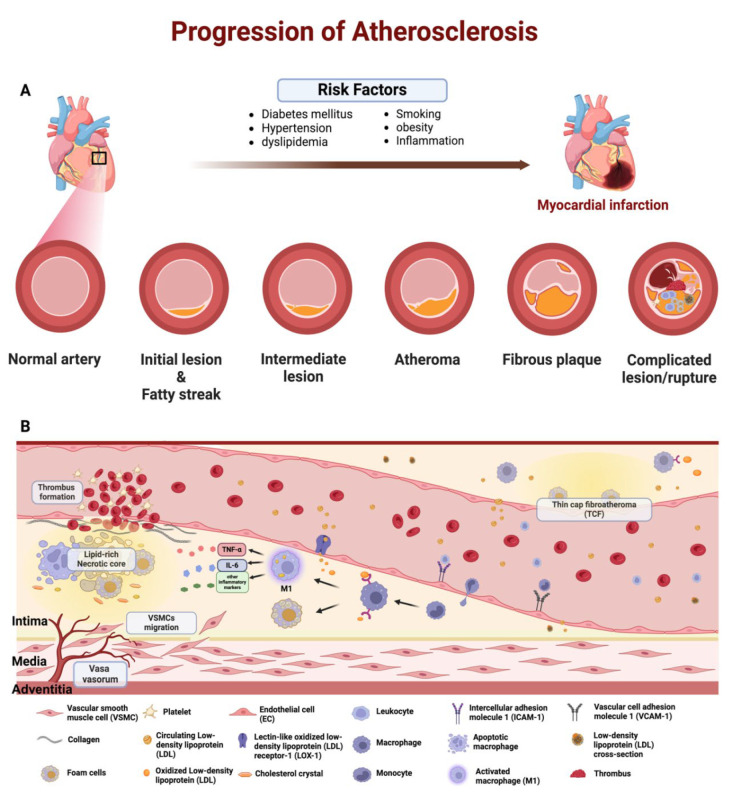
Schematic illustration of the progression of atherosclerosis. (**A**) The characteristic four pathological stages leading to the rupture of a complicated plaque lesion. (**B**) The development of atherosclerotic lesions involves the activation of macrophages and their subsequent foam cells, migration of smooth muscle cells (SMCs), and synthesis of extracellular matrix macromolecules such as collagen. Dead foam cells and SMCs form a lipid-rich necrotic core that presses on the endothelial cells creating thin cap fibroatheroma (TCF). Physical disruption of atherosclerotic plaque stimulates blood coagulation and ultimately results in thrombosis. Created with BioRender.com, accessed on 25 May 2024.

Atherosclerotic plaque deposition can be a non-resolving chronic inflammation that develops in the arteries and disturbs blood flow, resulting in cardiovascular diseases such as myocardial infarction, ischemic stroke, and peripheral artery disease [1,3]. The atherosclerotic plaque is made of a complex environment containing lipids, cholesterol crystals, many inflammatory cells such as monocytes, macrophages, and foam cells, and their secreted cytokines [4]. The atherosclerotic plaque can develop into either a stable or an unstable (vulnerable) plaque in which the vulnerable plaques have thin fibrous caps, are prone to rupture, and can subsequently cause thrombosis, obstructing the lumen and leading to acute ischemic events [5]. Multiple factors, including diseases like hypertension and hypercholesterolemia, damage vascular endothelial cells, leading to dysfunction, inflammation, and influencing atherosclerosis plaque formation, progression, and rupture (Figure 1) [6,7].

Dysregulation of lipid profiles, encompassing cholesterol, low-density lipoprotein (LDL), triglycerides (TGs), and high-density lipoprotein (HDL), coupled with monocyte infiltration into the intimal layer, initiates plaque development. This facilitates lipid deposition within the subendothelial space, leading to vascular inflammation and subsequent thrombogenesis [8]. The resident macrophages or monocyte-derived macrophages can proliferate within the damaged vessels and engulf oxidized-low-density lipoprotein (LDL) to become foam cells [9]. As a result, the structural integrity of macrophages is compromised and rather than facilitating tissue repair to preserve homeostasis, they adopt proinflammatory phenotypes that intensify the progression of atherosclerotic plaque development (Figure 1B). Following plaque formation, macrophages can also sustain plaque stability via phagocytosis, eliminating apoptotic cells to avoid plaque disruption [10].

During the advanced stages of plaque development, the extracellular matrix (ECM) acts as a conducive environment for the accumulation of lipids and cholesterol crystals, as well as the formation of microvessels, leading to the establishment of a necrotic core surrounded by a fibrous cap within the plaque [10]. The consequent thinning of the fibrous cap, coupled with an expansion of the necrotic core, escalates the risk of thrombosis, potentially triggering myocardial infarction (MI) [11].

Diagnosing atherosclerotic plaques is crucial for preventing cardiovascular events, and employs various imaging modalities, each with inherent advantages and limitations. Non-invasive techniques, such as ultrasound, computed tomography (CT), magnetic resonance imaging (MRI), and positron emission tomography (PET) are instrumental in detecting and characterizing plaques [12]. Ultrasound, including carotid intima-media thickness measurements, offers a cost-effective, initial assessment tool but lacks the specificity and depth of information provided by more advanced imaging techniques [13]. CT, especially coronary CT angiography (CCTA), provides high-resolution images of coronary calcification and plaque structure; however, it exposes patients to ionizing radiation and may require nephrotoxic contrast agents, limiting its use in certain populations [12]. MRI offers detailed insights into plaque composition without radiation exposure, yet its utility is constrained by longer acquisition times making it susceptible to motion artifacts that result from phase gain and loss, higher costs, and contraindications in patients with certain implants [12]. PET, often combined with CT or MRI, excels in identifying metabolically active, potentially vulnerable plaques through inflammation imaging, but its application is limited by availability, cost, and the need for radioactive tracers [14]. Despite these technological advances, a key limitation across all modalities is the difficulty in predicting plaque rupture, the most common cause of acute coronary events [14]. Furthermore, discrepancies in accessibility, patient compatibility, and the requirement for specialized equipment and expertise underscore the need for individualized diagnostic approaches and ongoing development of more predictive, universally accessible, and non-invasive diagnostic tools for atherosclerosis.

Despite significant advancements in understanding the risk factors associated with cardiovascular disease (CVD) and the widespread use of lipid-lowering drugs, CVD remains the leading cause of mortality globally [15]. Between 1990 and 2019, the period witnessed a 4.6% reduction in ischemic heart disease cases, primarily attributable to improved healthcare strategies and heightened awareness of cardiovascular risks [16]. Similarly, the years from 2011 to 2017 saw a 2.7% annual decrease in the mortality rate from coronary heart disease, reflecting the enhanced efficacy of contemporary therapeutic approaches, including statins and other cholesterol-managing medications [17]. These trends underscore the positive impact of targeted interventions and public health initiatives to reduce the burden of CVD. However, the persistent prevalence of CVD as the foremost cause of death worldwide highlights the complex interplay of genetic, environmental, and socioeconomic factors that contribute to its incidence. This continued dominance of CVD as a leading health challenge emphasizes the necessity for innovative prevention, diagnosis, and treatment strategies, including personalized medicine, advanced imaging techniques, and novel therapeutics, to further diminish its global impact [18].

Nanotherapeutic strategies have shown significant potential, particularly with the employment of polymeric materials, such as poly(d-lactic acid), polyethylene glycol (PEG), and poly lactic-co-glycolic acid (PLGA). These materials are noted for their biodegradability and ability to facilitate controlled release mechanisms, thereby acting as effective vehicles for the precise transport of biomolecules and proteins to atherosclerotic lesions [19]. The NP specificity enhances this approach, allowing for anti-inflammatory agents’ targeted and localized administration. Examples of such targeted delivery include the conveyance of the Ac2-26 peptide or the interleukin-10 cytokine directly to the sites of plaque formation, illustrating the capability of this method to precisely mitigate the inflammatory processes involved in plaque development [19]. Furthermore, nanoparticles (NPs) with anti-collagen IV ligands have enhanced fibrous cap thickness while diminishing the necrotic core area within atherosclerotic lesions in mouse models [20]. This dual action can mitigate the inflammatory response and stabilize the plaque’s structural integrity, advancing the nanotherapeutic modulation of atherosclerotic disease progression [20].

**Figure 2:**
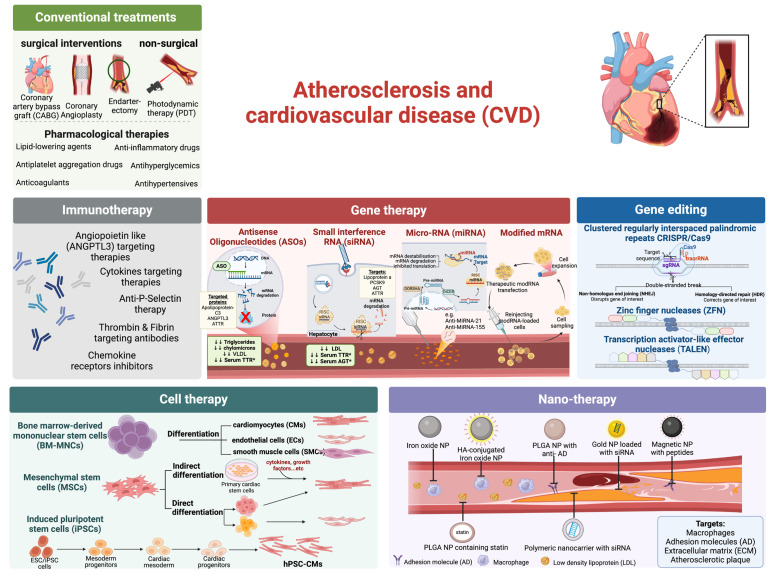
Graphic summary of the conventional and emerging therapies in atherosclerosis. Created with BioRender.com, accessed on 25 May 2024.

The evolving landscape of CVD management is on the cusp of a significant transformation, highlighted by breakthroughs in nanomedicine, advanced biomaterials, immunotherapeutic strategies, cell-based therapies, and genetic interventions. These innovative approaches collectively represent a paradigm shift towards more targeted, efficient, and personalized treatment modalities. Nanotechnology is set to refine therapeutic delivery and diagnostic precision through NP-mediated targeting of atherosclerotic lesions, promising enhanced specificity and reduced systemic toxicity. The advent of novel biomaterials is apparent in cardiac tissue engineering and regenerative medicine, enabling the development of bioresorbable vascular scaffolds and biocompatible platforms for tissue regeneration. Extending its reach beyond oncology, immunotherapy offers a path for reducing inflammatory keystones of atherosclerosis through targeted modulation of immune responses [21]. Cell therapy, leveraging the regenerative capacity of stem and progenitor cells, aims to restore cardiac function post-myocardial infarction by promoting the repair and regeneration of damaged myocardium [22]. Meanwhile, gene therapy, empowered by precision gene-editing tools like Clustered Regularly Interspaced Short Palindromic Repeats (CRISPR), offers the potential for direct correction of genetic determinants of CVD and the therapeutic modulation of gene expression to promote vascular repair and regeneration [23]. This article critically examines these emerging technologies, focusing on their underlying mechanisms, clinical applicability, and the challenges that must be navigated to realize their full potential in revolutionizing CVD management. Figure 2 summarizes the conventional and emerging therapies for atherosclerosis.

## 2. Atherosclerotic Treatment

Atherosclerosis is a complex disease characterized by the deposition of plaques in arterial walls, leading to the narrowing and stiffening of the blood vessels. Pharmacotherapy plays a pivotal role in the management of atherosclerosis by targeting various underlying mechanisms, including lipid metabolism, platelet aggregation, blood pressure control, and inflammation. Different drug classes are used in the treatment of atherosclerosis.

### 2.1. Lipid Metabolism Drugs

Statins, including atorvastatin, simvastatin, and rosuvastatin, are widely regarded as the fundamental elements in the therapeutic management of atherosclerosis. These agents function as inhibitors of 3-hydroxy-3-methylglutaryl-coenzyme A (HMG-CoA) reductase, an enzyme that governs the critical step in cholesterol biosynthesis. The principal effect of statins is the reduction in levels of low-density lipoprotein cholesterol (LDL-C). Extensive evidence consistently demonstrates their effectiveness in diminishing the occurrence of cardiovascular events and mortality among individuals afflicted with atherosclerotic CVDs [24]. The guidelines issued by the American College of Cardiology (ACC), American Heart Association (AHA), and European Society of Cardiology (ESC) recommended statin therapy as the primary intervention for individuals affected by atherosclerosis or those at a heightened risk of cardiovascular complications [25,26].

Cholesterol absorption inhibitors like Ezetimibe act by targeting the Niemann–Pick C1-Like 1 (NPC1L1) protein within the small intestine’s brush border. This action obstructs the uptake of cholesterol from the diet, leading to decreased levels of LDL-C. For enhanced lowering of LDL-C, Ezetimibe is often used alongside statins [27].

*PCSK9* inhibitors represent a newer category of medications that significantly reduce LDL-C by targeting the proprotein convertase subtilisin/kexin type 9 (*PCSK9*). This protein typically breaks down LDL receptors in the liver. By impeding *PCSK9’s* function, these drugs boost the number of available LDL receptors, which in turn accelerates the removal of LDL-C from the blood. *PCSK9* inhibitors, like evolocumab, are pivotal in atherosclerosis management, especially when statins are insufficient. Studies, such as Schwartz et al.’s 2020 landmark trial, underscore their efficacy in reducing LDL-C and cardiovascular risk, marking a significant advancement in treating hypercholesterolemia and preventing cardiac events [28].

Inclisiran is a novel therapeutic agent that has shown promise in the treatment of atherosclerosis, particularly in reducing LDL-C levels. It belongs to a class of medications known as small interfering RNA (siRNA) therapies, which target specific genes involved in cholesterol metabolism. Inclisiran operates by impeding the synthesis of the *PCSK9* protein, which is crucial for controlling the amount of LDL receptors in liver cells. By diminishing *PCSK9*, nclisiran boosts the number of LDL receptors, resulting in improved removal of LDL-C from the circulation. Furthermore, the ORION trials investigated the effectiveness and safety of inclisiran in individuals with atherosclerotic CVD or familial hypercholesterolemia. The outcomes from these trials have shown that inclisiran consistently achieves substantial decreases in LDL-C levels, which could lead to a decrease in cardiovascular events [29].

Previous studies on fibrates have focused on their potential to manage atherosclerosis, especially for patients with dyslipidemia who exhibit high triglyceride levels or mixed dyslipidemia. The mechanism of action for fibrates involves the activation of peroxisome proliferator-activated receptors, which is PPAR-alpha. This activation alters the expression of genes that are critical to lipid metabolism, leading to lowered triglyceride levels and increased levels of HDL cholesterol. Treatment with fibrates targeting signs of atherogenic dyslipidemia significantly lowers the risk of future vascular incidents [30]. Fibrates can act as activators of the peroxisome proliferator-activated receptor (PPAR) alpha and could be utilized in managing dyslipidemia. Their primary function is to decrease the level of TGs and LDL, and at the same time, raise the levels of HDL. Individuals with chronic kidney disease (CKD) exhibit a unique lipid profile marked by increased levels of triglyceride-rich lipoproteins and reduced HDL levels. This lipid imbalance is linked with the early stages of atherosclerosis, coronary artery disease (CAD), and increased risk of death.

### 2.2. Antiplatelet Aggregation Drugs

Antiplatelet agents, such as aspirin and P2Y12 inhibitors, are considered the cornerstones in preventing atherothrombotic events in atherosclerosis [31]. They can inhibit platelet activation and reduce the formation of blood clots within atherosclerotic arteries, thereby mitigating the risk of heart attacks and strokes. Aspirin irreversibly inhibits cyclooxygenase-1, reducing the production of thromboxane A2, a potent platelet activator. Clopidogrel, ticagrelor, and prasugrel selectively inhibit the P2Y12 receptor on platelets, which in turn, would prevent the platelet activation and aggregation. Aspirin is widely recommended as a first-line antiplatelet therapy in atherosclerosis due to its proven efficacy in reducing cardiovascular events [32].

### 2.3. Antihypertensive Drugs

Antihypertensive medications are widely used in the management of atherosclerosis, as high blood pressure is considered a critical risk factor for the development and progression of this disease. Such medications work by lowering the systemic blood pressure, thus reducing the mechanical stress on arterial walls and slowing the atherogenic process. Different classes of antihypertensive drugs, including angiotensin-converting-enzyme (ACE) inhibitors, angiotensin II receptor blockers (ARBs), calcium channel blockers, and beta-blockers, offer protective effects on the vasculature.

ACE inhibitors not only lower blood pressure but also possess anti-inflammatory properties that stabilize atherosclerotic plaques [10]. ACE inhibitors stabilize atherosclerotic plaques primarily by reducing the production of angiotensin II, a key driver of vasoconstriction, inflammation, and oxidative stress that contribute to plaque instability. By inhibiting the conversion of angiotensin I to angiotensin II, these drugs not only decrease vascular inflammation but also improve endothelial function through enhanced availability of nitric oxide, a crucial vasodilator and anti-inflammatory agent [33]. This improvement in endothelial function further helps in reducing arterial stiffness and maintaining vascular integrity. Additionally, the reduction in angiotensin II levels leads to decreased oxidative stress, which protects the endothelium and reduces the risk of plaque rupture. Collectively, these mechanisms make ACE inhibitors effective in promoting plaque stability and preventing cardiovascular events [34]. ARBs similarly decrease the action of angiotensin II, leading to vasodilation and reduced vascular inflammation. Calcium channel blockers improve endothelial function and reduce arterial stiffness, while beta-blockers decrease the myocardial oxygen demand and can limit the progression of CAD.

Overall, the strategic use of antihypertensive medications can attenuate the progression of atherosclerosis and reduce the incidence of cardiovascular events such as myocardial infarction and stroke. The choice of antihypertensive therapy is typically individualized, taking into account the patient’s overall cardiovascular risk profile and comorbid conditions.

### 2.4. Anticoagulant Drugs

Anticoagulant medications play a vital role in managing atherosclerosis, particularly in the prevention of thrombotic events that can arise from atherosclerotic plaques. These drugs prevent blood clot formation, crucial for lowering the risk of stroke and heart attacks in individuals with atherosclerosis. Anticoagulants may offer dual benefits in atherosclerotic care by both stabilizing plaques and reducing the likelihood of clot formation, potentially offering a combined approach to the treatment when used alongside antiplatelet agents [35].

### 2.5. Sugar Metabolism Drugs

Antihyperglycemic drugs like metformin, exert their beneficial effects on atherosclerosis through various mechanisms. They reduce insulin resistance, lower blood glucose levels, and inhibit hepatic glucose production. Metformin also possesses anti-inflammatory properties and can hinder the proliferation of smooth muscle cells involved in atherosclerotic plaque formation [36].

Sodium–glucose cotransporter-2 (SGLT2) inhibitors, another group of antihyperglycemic drugs, may impact atherosclerosis through distinct mechanisms. By blocking glucose reabsorption in the kidneys, they increase urinary glucose excretion and lower blood glucose levels. SGLT2 inhibitors can also improve endothelial function, reduce oxidative stress, and decrease arterial stiffness, and hence, contribute to the prevention and regression of atherosclerosis [37].

Moreover, the lab-created peptide, tirzepatide, serves as a dual agonist for both glucose-dependent insulinotropic polypeptide (GIP) and glucagon-like peptide-1 (GLP-1) receptors, which may explain its significant impact on lowering both glycated hemoglobin and body weight in type 2 diabetes mellitus patients. Not only does it help manage blood sugar levels and reduce obesity, but it may also offer some cardiovascular benefits. The combined targeting of GIP and GLP-1 by tirzepatide was linked to synergistic reductions in weight, appetite, and fat levels, with these effects verified in human subjects as well as animal studies. However, more research is necessary to fully explore the long-term cardiovascular implications of tirzepatide treatment [38].

### 2.6. Cytokine-Targeting Therapy

Cytokines are a group of small, secreted proteins released by various cells in the body that are involved in the communication and interaction between cells in several signaling pathways [39]. Given the involvement of cytokines in the inflammation process, targeting inflammatory cytokines as a strategy to suppress inflammation in atherosclerosis has been investigated [40,41]. The formation of atherosclerotic plaques involves several cells, adhesion molecules, and the activation of inflammatory cytokines, including interleukin-1 beta (IL-1β), tumor necrosis factor-alpha (TNF-α), interleukin-6 (IL-6), interleukin-17 (IL-17), and interferon gamma (IFN-γ), which were identified as potential therapeutic targets of atherosclerosis [39,41,42]. IL-1β, in addition to TNF-α, serves as a central regulator of endothelial cell activation and promotor of cellular recruitment to inflammatory sites [42].

Given the involvement of multiple inflammatory pathways in atherosclerosis, several clinical trials examined the effects of anti-inflammatories in atherosclerosis and demonstrated positive results [40,41,43]. Several anti-inflammatory agents were investigated for their potential therapeutic benefits in the management of this condition. Canakinumab functions as a monoclonal antibody (mAb) with a specific affinity for IL-1β, a cytokine that drives inflammation. IL-1βs’ role in atherosclerosis is significant, as it not only activates endothelial cells but also draws in and stimulates immune cells within the plaque regions. Canakinumab effectively reduces the activity of IL-1β. This blockade of IL-1β helps to diminish inflammatory responses in atherosclerotic plaques, potentially contributing to their stabilization and a lowered incidence of cardiovascular events [44]. The canakinumab Anti-inflammatory Thrombosis Outcomes Study (CANTOS) is the first trial to confirm the notion that targeting inflammation could be beneficial in the management of atherosclerosis. This study targeted canakinumab, the anti-IL-1β antibody, in a randomized clinical trial of patients with stable CVD after a previous myocardial infarction, with an elevated C-reactive protein (CRP) and was being treated according to current guideline recommendations. This trial resulted in a significant risk reduction in the recurrence of major adverse cardiovascular events (MACE), confirming that targeting inflammatory pathways is of clinical importance and provides a beneficial effect in the management of atherosclerotic CVD [44]. Nevertheless, the risk–benefit ratio of using an anti-IL-1β antibody warrants attention, as patients who received canakinumab in the CANTOS trial were at a higher risk of developing fatal infections. Such findings may help develop anti-inflammatories with better safety profiles that can be studied and evaluated for the management of atherosclerosis [40,41,44].

Furthermore, IL-1 blockade using anakinra, an IL-1 receptor antagonist, in patients with ST-segment-elevation myocardial infarction (STEMI) was also evaluated in the Virginia Commonwealth University Anakinra Remodeling Trial 3 (VCUART3). It was found that anakinra can reduce the area under the curve of high-sensitivity CRP (hs-CRP), supporting the potential benefit of IL-1 blockade in acute coronary syndrome (ACS) [45].

Similarly, IL-6 is a cytokine deeply involved in atherosclerosis. Targeting IL-6 has emerged as a promising strategy for curtailing inflammation and enhancing cardiovascular health. Tocilizumab, an antibody that inhibits the IL-6 receptor, is typically utilized to manage autoimmune diseases, including rheumatoid arthritis, and may also offer benefits in atherosclerosis management. Tocilizumab has the potential to slow down the beginning and development of atherosclerosis, as well as to fortify atherosclerotic plaque [46]. In the phase 2 clinical trial of patients with chronic kidney disease and high hsCRP (≥2 mg/L), inhibition of IL-6 using ziltivekimab, an anti-IL-6 mAb, reduced hsCRP by 96.2% compared to a placebo, providing further evidence of the potential benefits of targeting inflammation in reducing atherosclerotic risk [47].

In addition, methotrexate, known for its application in treating rheumatologic diseases, was explored for its anti-inflammatory effects in atherosclerosis management. The premise was that methotrexate might mitigate the advancement of atherosclerosis and its associated cardiovascular risks by dampening systemic inflammation. Nonetheless, outcomes from the Cardiovascular Inflammation Reduction Trial (CIRT) indicated that methotrexate did not reduce cardiovascular events in patients with a history of atherosclerosis, casting doubt on its efficacy for cardiovascular prevention in this setting [48]. Moreover, results from studies on the use of colchicine, an anti-inflammatory that inhibits tubulin polymerization and limits NOD-like receptor protein 3 (NLRP3) inflammasome activity in CAD also demonstrated beneficial effects in reducing major adverse cardiovascular events [39,49].

### 2.7. Anti-P-Selectin Therapy

P-selectin is an adhesion molecule expressed on activated platelets and has a key role in atherosclerosis, as it binds to monocytes, neutrophils, and T-cells, enabling the first steps of leukocyte extravasation and accelerating plaque formation [50,51]. Studies have demonstrated that soluble P-selectin has prothrombotic and procoagulant properties, which places P-selectin as an intriguing therapeutic target in atherosclerosis [52]. Pre-clinical studies on animal models have suggested that inhibiting P-selectin can decrease immune cells and platelet adhesion after myocardial injury [53,54].

One of the pharmacotherapies targeting P-selectin is inclacumab, a recombinant mAb against P-selectin, which was evaluated in the SELECT-ACS (Effects of the P-Selectin Antagonist Inclacumab on Myocardial Damage after Percutaneous Coronary Intervention for Non-ST-Elevation Myocardial Infarction) trial. It was demonstrated that administering inclacumab at a single infusion dose of 20 mg/kg before percutaneous coronary intervention (PCI) significantly reduced myocardial damage (defined as the change in troponin and creatine kinase from baseline) after PCI [55]. However, this study only included patients with non-ST elevation myocardial infarction who underwent PCI. Whether this benefit extends to patients presenting with STEMI (i.e., ST-elevated myocardial infarction) or to patients who may not undergo PCI remains unknown. Thus, future studies evaluating its use in patients with other types of ACS (i.e., STEMI), and in patients who may not undergo PCI, are needed to provide further insight into its clinical use in atherosclerotic CVD.

### 2.8. Angiopoietin like (ANGPTL3) Targeting Agents

Angiopoietin-like proteins (ANGPTL) are secretory glycoproteins that play an important role in angiogenesis and are composed of a family of eight proteins (ANGPTL 1–8) [56]. Genetic studies have identified mutations, single nucleotide polymorphisms (SNPs), around the *ANGPTL3* locus that were associated with altering lipid function and metabolism [56,57]. Loss of function mutations of *ANGPTL3* genes resulted in lower risk for CAD and lower plasma levels of LDL, TGs, and total cholesterol [58,59,60]. These findings instigated the development of novel pharmacological agents that target *ANGPTL3* inhibition.

Evanicumab is a human IgG4 mAb that inactivates *ANGPTL3*, preserving the function of lipoprotein lipase (LPL) and endothelial lipase (EL) that are responsible for the breakdown of TGs in very-low-density lipoprotein (VLDL), and chylomicrons [61]. Initially, evanicumab was studied in patients with homozygous familial hypercholesterolemia (HoFH) in the Evinacumab Lipid Studies in Patients with Homozygous Familial Hypercholesterolemia (ELIPSE HoFH) trial. It was demonstrated that at the 24th week, intravenous infusion of evinacumab (15 mg/kg every 4 weeks) resulted in a 47.1% reduction in the baseline of LDL as compared to an increase of 1.9% in the placebo group, in patients with HoFH on maximum doses of lipid-lowering therapy [62]. Similar results were also found when evinacumab was evaluated in patients with refectory hypercholesteremia who were on maximum tolerated doses of lipid-lowering therapies [63].

Another pharmacological agent that targets *ANGPTL3* is vupanorsen, an antisense oligonucleotide that targets hepatic *ANGPTL3mRNA* and inhibits ANGPTL3 protein synthesis [64]. Vupanorsen was studied in patients with hypertriglyceridemia (>150 mg/dL), type 2 diabetes mellitus, and hepatic steatosis. It was found to significantly reduce TGs, apolipoprotein C-III, HDL cholesterol, remnant cholesterol, and total cholesterol, but not LDL, when compared to a placebo (26). Furthermore, the use of vupanorsen reduced the non–HDL cholesterol (non-HDL-C) in the TRANSLATE (Targeting *ANGPTL3* with an Antisense Oligonucleotide in Adults with Dyslipidemia)–TIMI (Thrombolysis in Myocardial Infarction) 70 trial in patients with high non-HDL-C and hypertriglyceridemia treated with statins [65].

Collectively, these findings suggest that inhibition of *ANGPTL3* provides new opportunities for lowering lipids and managing residual cardiovascular risk, especially in patients at high risk with refractory dyslipidemia. Future, studies on the effects of *ANGPTL3* inhibition on atherosclerotic CVD outcomes are needed to fully understand the full potential of these novel pharmacological agents in the management of atherosclerosis.

### 2.9. Targeting Plaque-Weakening Proteases

Atherosclerotic CVD can be caused by stable or unstable atherosclerotic plaque formation. The unstable plaque, which is called vulnerable plaque, may lead to thrombosis owing to its ability to puncture the area surrounding the plaque, which will ultimately cause ischemia by inhibiting the blood supply. To target vulnerable plaques, it is important to understand the structure of these plaques and the behavior of their content. The main feature content of vulnerable plaque is the lipid core that is large and has a fibrous cap, i.e., collagen, highly inflamed, and thin. This combination is called a thin cap fibroatheroma (TCFA), which can cause inflammation of the surrounding area [66]. The vulnerable plaque might be remodeled and destabilized by proteases. Protease is an enzyme that can digest the ECM proteins, which in the case of vulnerable plaque, can weaken the fibrous cap and destabilize it, ultimately causing its rupture [67].

The high concentration of matrix metalloproteinases (MMPs) is known as an indicator for vulnerable plaque weakening. MMPs have a role in the degradation of elastin, fibrin, gelatine, collagen, and several other ECM proteins; yet they can support the smooth muscles’ migration and proliferation that has a vital role in improving the stability of the fibrous cap of the vulnerable plaque [67]. There are approximately 23 MMPs that have similar mechanisms since they share the same catalyst motif which is zinc ion that bands to histidine [68,69]. This catalyst motif targets its substrate by binding then cleavage of its target which is mainly cell surface proteins or ECM [69]. The concentration of MMPs can be high and can reach 50 nM in severe atherosclerosis [67]. From the structure of MMPs, many designated MMP inhibitors can be synthesized to target the zinc catalyst motif of MMPs. For instance, CGS 25966 and CGS 27023A are derivatives of N-sulphonyl amino acid hydroxamates that are highly potent against MMP-1, MMP-2, MMP-3, and MMP-9 by targeting the zinc catalyst motif [70].

### 2.10. Targeting Thrombin and Fibrin

Thrombin is a serine protease that in normal conditions has many biological roles such as angiogenesis, thrombogenesis, and repairing injured tissues. The inactive form of thrombin, which is found in the blood circulation, is called prothrombin. In atherosclerosis, the activation of platelets occurs and will interact with leukocytes and trigger the release of proinflammatory mediators such as reactive oxygen species and cytokines. This will cause interaction between tenase and prothrombinase which causes a conversion of prothrombin to thrombin and fibrin [71,72,73]. The formed fibrin can be accumulated in the plaque of the atherosclerotic patient that participates in the evolution of the disease [74,75].

The detection of thrombin can be performed by using a peptide ligand with a sequence of GPXRSGGGGKC, which can be cleaved by thrombin, and then the sequence could be linked to a Cy5.5 dye to detect and image thrombin in vivo [76]. Also, another cleavable peptide by thrombin that has a KKLVPRGSL sequence was used as a linker between iron oxide and gadolinium chelate layer for the detection of thrombin using MRI [77,78]. Regarding fibrin, two mAb were engineered by Raut et al. [79], which were named 1H10 and 5F3. Both antibodies can bind to different domain moieties of fibrin and fibrinogen. 1H10 has a stronger binding affinity to fibrin than 5F3 in the E domain. Another mAb that was named AP2 has a selective targeting ability to fibrin over fibrinogen by recognizing the N-terminal of the α-chain of fibrin [80].

### 2.11. Photodynamic Therapy (PDT)

Photodynamic therapy (PDT) is a technique that can be used for the treatment of several diseases including atherosclerosis. It consists of photosensitizer, light, and oxygen dissolved in the tissue being treated. The mechanism of PDT is that a photosensitizer is highly accumulated in the affected area such as cancerous cells and atherosclerotic lesions. Each photosensitizer can be activated at a specific wavelength using a specific light. This will lead to the destruction of specific diseased cells due to the interaction between the stimulated photosensitizers and the abnormal cells.

In atherosclerosis, PDT can be defined by porphyrin derivative photosensitizers. The uptake of hematoporphyrin by the atherosclerotic plaque toward the aortic wall was detected in an atherosclerotic rabbit model [81]. This study concluded that hematoporphyrin can prevent the growth of smooth muscle cells and decrease atheroma after approximately two weeks of therapy in comparison to the control group. However, the main limitations of using hematoporphyrin as a photosensitizer in clinical applications are that it only gets stimulated at 630 nm light which cannot cross the endoluminal blood at an adequate level and it can cause photosensitivity when applied subcutaneously [82,83].

A benzoporphyrin derivative, verteporfin, which is a second-generation photosensitizer, was used as a PDT in atherosclerosis [84]. It is effective against hyperlipidemic plaque due to its ability to interact with LDL and cause apoptosis after stimulation by light at 692 nm [85]. Verteporfin is a highly selective and potent photosensitizer in the removal of accumulated atherosclerotic plaque [84,86]. The Texaphyrin family which includes motexafin lutetium contains aromatic macrocycles, which resemble porphyrin but with a superior unique feature. The main advantage of motexafin lutetium is the presence of lutetium, a diamagnetic lanthanide (III) cation, which enhances the potency of motexafin as a photosensitizer to be stimulated at a higher absorption spectrum wavelength of >720 nm in comparison to porphyrins which have an absorption spectrum of 630 and 665 nm [87].

All the abovementioned emerging and targeted therapies hold a huge potential for identifying and developing novel pharmacological and diagnostic agents to effectively manage atherosclerosis and CVD. Further pre-clinical investigation and larger clinical trials of these agents focusing on cardiovascular outcomes are needed to better understand their role in the management of atherosclerotic CVD and to optimize their use in clinical practice.

## 3. Recent Advancements in Cell Therapy

Cell-based cardiac repair is a promising field in myocardial regeneration. Aside from medications and cardiac devices, stem cells have the potential to regenerate and mend damaged heart tissues [88]. Among potential cell therapies for cardiac repair, bone marrow-derived mononuclear stem cells (BM-MNCs) have emerged as a highly researched option. These versatile cells hold the potential to revitalize damaged heart tissues by generating new cardiomyocytes (CMs), endothelial cells, and smooth muscle cells. This ability to repopulate the infarct region, i.e., a scarred area after a heart attack, with functional cells, has shown promising results in restoring heart function. However, another contender in the race for cardiac regeneration is the mesenchymal stem cell (MSC). These cells, also found in bone marrow, exhibit remarkable potential for regenerating cardiac tissues. Their flexibility in differentiating into various cell types, including those crucial for heart function, makes them a valuable candidate for further research and development [89,90].

Several in vivo and in vitro pre-clinical models, as well as clinical studies for myocardial infarction and heart failure, have extensively examined MSCs, with varying results. MSCs constitute a good healing tool for CVD, including protection of the myocardium, reduction of inflammation, enhanced apoptosis resistance, prevention of fibrosis, and improved myocardial cell differentiation surrounding infarct sections, and angiogenesis. They have also proved to have substantial advantages in CVD treatment, but several barriers persist [91]. These are mainly involved in low survival rates in ischemic myocardium and bad-focused migration [92]. Clinical studies have shown the safety and practicality of MSC therapy; however, safety and long-term efficacy still need further investigation.

Endothelial progenitors facilitate the regeneration of the endothelial lining of blood vessels. They help repair the damaged lining of blood arteries after myocardial infarction through mobilization [91]. They have expressed great potentiality in several preliminary clinical studies for treating CVDs. Induced pluripotent stem cells (iPSCs) are derived by reprogramming adult somatic cells and they can develop into CMs [93]. Deriving patient-specific iPSCs that might evade the immune system is a significant advantage of this method, which bodes well for the new fields of regenerative medicine and personalized medicine.

Both iPSCs and human embryonic stem cells (hESCs) have been common topics of research in recent years since their exploitation led to the generation of CMs. Human pluripotent stem cell-derived CMs (hPSC-CMs) displayed molecular markers as well as subcellular structures and electrophysiological properties, similar to young, primary CMs [94]. Since then, it was demonstrated in animal studies that after myocardial infarction hPSC-CMs might potentiate contractile performance via engraftment, survival, and electrical coupling with host cardiac tissue [94]. Transplanted hPSC-CMs can survive and generate functional myocytes with a striated organization in rats with acute myocardial infarction and chronic post-infarction heart disease [95,96]. After an acute myocardial infarction, these hPSC-CM injections reduced ventricular dilation and preserved systolic function. Autologous cardioplasty with clinical-grade hPSC-CM patches was performed on a Japanese male patient with ischemic cardiomyopathy’s severe heart failure. Six months after the intervention, the clinical symptoms appeared to be rectified with insignificant side effects and no modification to the transplant site and wall motion of the heart [97]. These first human clinical studies show that treatment of heart injury with hPSC-CMs seems promising.

However, there are several cell-based cardiac repair limitations. Low engraftment of therapeutic cells in cardiac tissue is a major problem. Transplanted cells have a poor prognosis when placed in cardiac tissue, reducing the efficacy of treatment [98]. The methods of tracking the migration, differentiation, and survival of transplanted cells in vivo are also lacking improvement [99]. There is a risk of arrhythmic problems following transplantation as a well-known conduction pathway of the heart may be disrupted by introduced cells [98]. It is also difficult to regulate the cells’ actions after transplantation, including their differentiation and proliferation. It is usually not practicable and often dangerous to extract a significant number of cardiac cells from a patient’s heart tissue [99]. Finally, adult mature CMs vary in functional features from human iPSCs [98]. These variations make accurate medication screening and practical therapeutic applications challenging.

The techniques used for regeneration may include a wide range of approaches. This is achieved through microfabrication that is used to fine-tune the cellular microenvironment, and thus, affects the generation and functionality of human iPSC-CMs. Signaling molecules used during culturing can be changed to affect the growth environment and yet, guide and stimulate the maturation of these cells. For human iPSC-CMs to achieve the same types of characteristics and capacities as adult CMs, long-term culturing hastens their maturation [100]. This procedure is commonly accompanied by additional approaches to ensure the highest possible efficiency of the treatment. Three-dimensional (3D) culture has the potential to improve the development and maturation of human iPSC-CMs in a more in vivo cardiac microenvironment nature [101]. The process of maturation and functional development of human iPSC-CMs may be directed by mechanical loading and electrical stimulation as a way of mimicking the characteristics of fully developed CMs [101]. The stiffness can be regulated to run and boost the maturation of human iPSC-CMs to the substrate. Finally, neurohormonal variables may also influence the function and maturation of human iPSC-CMs [102].

## 4. Recent Advancements in Gene Therapy

Gene therapy has emerged as a transformative frontier in cardiovascular medicine, offering innovative approaches to address complex conditions such as CVD [103]. Various molecular tools have been developed within this realm to modulate gene expression, paving the way for precise therapeutic interventions. Antisense oligonucleotides (ASOs), siRNA, microRNA, and modified mRNA represent cutting-edge technologies that hold immense potential in reshaping the landscape of CVD treatment [104]. These sophisticated tools enable researchers to target specific genes, modulate gene expression, and intervene at the molecular level, offering promising strategies to mitigate the underlying genetic factors contributing to CVD [105].

### 4.1. Antisense Oligonucleotide (ASO)

Recent advancements in RNA-targeted ASO therapeutics for CVD showcase innovative progress. This approach uses advanced nucleic acid chemistry to create entirely synthetic “nucleic acid drugs”, offering a promising solution for addressing intricate molecular mechanisms in CVD [106]. The most advanced approach involves N-acetyl galactosamine (GalNAc) coupling of the nucleic acid, directing it to asialoglycoprotein receptors (ASGPR) on hepatocytes. This strategy significantly enhances the potency of the drug, particularly evident in the context of ASOs [107]. ASOs, consisting of single-stranded DNA and typically ranging from 13 to 20 nucleic acids, work to downregulate the expression of a specific molecular target. This is achieved by binding to mRNA, forming a duplex cleaved by RNAse H1, leading to the inhibition of protein synthesis [108,109].

#### 4.1.1. ASO Targeting Apolipoprotein C-III

RNA-targeted ASOs have demonstrated significant potential in pivotal cardiovascular trials, particularly those targeting Apolipoprotein C-III (ApoC-III) [110]. ApoC-III inhibits lipoprotein lipase (LPL) and delays the clearance of triglyceride-rich lipoproteins, contributing to increased cardiovascular risk. Clinical trials of the first generation of volanesorsen, an ASO targeting ApoC-III, including APPROACH (NCT02211209) and COMPASS (NCT02300233), have confirmed the efficacy of volanesorsen in reducing TG levels by up to 77% and 71.8%, respectively, with potential benefits for patients with hypertriglyceridemia [111]. While thrombocytopenia was observed as a side effect, ongoing research with modified ASOs, such as GalNAc-conjugated ones, aims to address these concerns and enhance the clinical utility of ApoC-III-targeting ASOs, potentially offering a safe and efficient approach for reducing TG levels in CVD, including familial chylomicronemia syndrome (FCS) [112,113]. GalNAc-volanesorsen, approved in the European Union (EU) for FCS, effectively lowers the TG levels by 78%, mitigating the risk of acute pancreatitis [112,114]. Clinical studies, such as NCT03385239, have explored GalNAc-conjugated volanesorsen targeting ApoC-III, showing promising outcomes in reducing atherogenic lipid profiles and improving safety profiles [115]. Furthermore, like volanesorsen, experimental studies of olezarsen, targeting ApoC-III, have demonstrated significant reductions in TG levels in animals and humans [116].

#### 4.1.2. ASO Targeting Angiopoietin-like 3

Angiopoietin-like 3 (ANGPTL3), a protein primarily synthesized in the liver, plays a vital role in inhibiting LPL and endothelial lipase, making it a novel genetic target for CVD risk reduction [117]. Loss-of-function mutations in *ANGPTL3* are associated with familial hypolipidemia, characterized by significantly low levels of LDL-C, TGs, and reduced CVD risk [118]. In a phase 1 study, a GalNAc-modified ASO successfully replicated the lipid phenotype of patients with familial combined hypolipidemia, demonstrating potential therapeutic mimicry [118,119]. Animal models further supported the efficacy of *ANGPTL3* inhibition, showing reductions in liver TGs and atherosclerosis progression alongside increased insulin sensitivity [120]. Notably, *ANGPTL3* inhibition substantially decreased VLDL-C, non-HDL-C, ApoB, and ApoC-III. A phase 2 trial (NCT03371355) explored the clinical safety of *ANGPTL3* inhibition [119]. Another study on GalNAc-conjugated mouse *ANGPTL3* ASOs demonstrated efficacy in mouse models, reducing ApoB-containing lipoproteins and improving insulin sensitivity [121]. A phase 1 trial in humans showed dose-dependent lipid reductions, with multiple administrations achieving a maximum 63% reduction in TGs and 36.6% reduction in non-HDL-C levels. Importantly, no serious side effects were observed [122]. While these findings suggest the potential of IONIS-*ANGPTL3* in reducing ApoB-containing lipoproteins, larger clinical trials linking *ANGPTL3* ASOs with cardiovascular outcomes are warranted for comprehensive evaluation.

#### 4.1.3. ASO Targeting Transthyretin Amyloidosis

Transthyretin amyloidosis (*ATTR*), impacting both the nervous system and heart, results from the abnormal accumulation of transthyretin (TTR), causing rare but significant morphological and functional changes in tissues [123]. *ATTR* can be wild-type (*wtATTR*) or mutated/variant (*vATTR*), with the latter associated with inherited *TTR* gene mutations [124]. Despite being traditionally considered rare, *ATTR*-cardiomyopathy is increasingly recognized as a noteworthy contributor to heart failure, particularly in older adults with preserved ejection fraction (HFpEF) [125]. Inotersen, a 2′-O-methoxyethyl-modified ASO targeting both wild-type and mutant *TTR* in the liver, demonstrated positive outcomes in a 15-month phase 3 study for hereditary *TTR* amyloidosis polyneuropathy [126]. Notably, inotersen lowered *TTR* levels in a dose-dependent manner, achieving a maximum *TTR* knockdown of 96% at 300 mg [127]. Eplontersen, a GalNAc-conjugated ASO, exhibited superior potency compared to inotersen. In a phase 1 study, eplontersen achieved a maximum *TTR* knockdown of 86% at a dose of 120 mg with rapid absorption. Safety profiling indicated good tolerability, paving the way for a potential clinical approval [124].

### 4.2. Small Interfering RNA (siRNA)

The role of small double-stranded RNA (dsRNA) therapeutics, including miRNAs and siRNAs, was pivotal in experimental studies and clinical trials [128]. The delivery of these therapeutics has evolved from early lipid-mediated transfection reagents to more advanced formulations [129]. The discovery of siRNA, a potent and specific gene-silencing mechanism mediated by double-stranded RNA, rapidly transitioned from fundamental research to successful clinical studies [130]. siRNAs, whether chemically synthesized or recombinant shRNAs, were explored for their ability to trigger RNAi and harness the RNA-induced silencing complex (RISC) molecular apparatus [131]. Recent clinical trials have underscored the efficacy of advanced siRNAs upon receptor-mediated cellular entry, especially when engineered to mitigate immunogenicity and enhance stability. The resulting stable complex with RISC leads to long-term efficiency, with siRNAs achieving up to six months of effective cleavage of target transcripts and subsequent suppression of encoded proteins [132].

#### 4.2.1. siRNA Targeting Proprotein Convertase Subtilisin/Kexin Type 9 (*PCSK9*)

In the cardiovascular field, the ORION clinical trials program currently stands as a forefront initiative, rigorously exploring RNAi-mediated *PCSK9* inhibition across diverse CVDs [133,134]. The published results of the ORION-1 trial (NCT02597127) highlight the remarkable impact of the siRNA drug inclisiran on *PCSK9*, showcasing profound reductions in LDL-C levels at day 180, with the two-dose 300-mg regimen achieving the greatest reduction (52.6%) [135]. The persistence of reduced *PCSK9* and LDL-C observed over 240 days following a single ORION-9 dose, underscores the potential of RNAi-mediated *PCSK9* inhibition in the liver as an alternative to mAb targeting of circulating *PCSK9*, with the added benefit of a likely reduced injection burden [136]. The subsequent ORION-10 and ORION-11 studies, conducted over 18 months, reaffirmed inclisiran’s effectiveness, showcasing a reduction in LDL-C by 52.3% and 49.9%, respectively, at day 510 [137]. The recently completed phase 3 clinical trial of ORION-4 further investigated cardiovascular outcomes related to siRNA *PCSK9* inhibition [138].

#### 4.2.2. siRNA Targeting Transthyretin Amyloidosis

The past decade has witnessed the development of several pharmacological therapies for *ATTR* amyloidosis [139]. ONPATTRO (formally known as Patisiran), the pioneering commercialized siRNA, obtained FDA approval in August 2018 [140]. In contrast to GalNAc-conjugated counterparts, ONPATTRO employs a unique liver uptake mechanism [141]. Formulated as lipid NPs (LNPs), ONPATTRO shields therapeutic oligonucleotides from endogenous enzyme degradation. The particle, opsonized by apolipoprotein E (ApoE) derived from circulating lipoproteins, enters the liver through vascular fenestrations and binds to LDLR on hepatocytes [142,143]. In phase 1 and 2 studies, ONPATTRO demonstrated a safe and effective reduction in serum TTR [144]. The phase 3 APOLLO trial focused on patients with *ATTR*-polyneuropathy, revealing positive outcomes for 126 patients with associated cardiac involvement [140]. Additionally, the treated group experienced a 46% reduction in hospitalizations due to cardiovascular causes and all-cause death compared to the placebo group [123]. Real-life evidence further supported ONPATTRO’s efficacy, including its positive impact on extracardiac symptoms such as gastrointestinal involvement [123].

#### 4.2.3. siRNA Targeting Lipoprotein(a)

Lipoprotein(a), or Lp(a), is linked to an elevated risk of ischemic heart disease and plays a causal role in atherosclerosis and calcific valvular aortic stenosis [145,146,147]. Its expression is regulated by the apolipoprotein(a) gene (*LPA*), and its levels are largely genetically determined [148]. Olpasiran, the first siRNA designed to target apolipoprotein(a) (ApoA) mRNA, demonstrated dose-dependent and sustained reductions in Lp(a) levels up to 6 months in phase 1 studies [149]. In the phase 2 OCEAN(a)-DOSE trial, olpasiran significantly reduced serum Lp(a) levels in a dose-dependent manner over 48 weeks, for the 225 mg dose administered every 12 weeks [134]. Another siRNA, lepodisiran, designed to reduce ApoA mRNA, showed promising results in a phase 1 study, with a dose-dependent reduction in serum Lp(a) concentrations up to 97% at a high dose of 608 mg [150].

#### 4.2.4. siRNA Targeting Angiotensinogen

Angiotensinogen (AGT), predominantly produced by the liver, has garnered attention as a potential target for innovative antihypertensive therapies [151]. Zilebesiran, the first siRNA designed to reduce AGT mRNA in hepatocytes, demonstrated liver-specific effects in pre-clinical studies and a phase 1 trial [152]. Notably, a four-part phase 1 study revealed clinically significant reductions in systolic and diastolic BP with single doses of zilebesiran, persisting for up to 24 weeks. Serum AGT reduction exceeded 90%, showcasing the drug’s efficacy. Safety and tolerability were affirmed, with fewer serious adverse events reported in zilebesiran-treated patients compared to the placebo group [153]. These advancements hold the potential to address therapeutic adherence issues and provide effective, long-term control for hypertensive patients.

### 4.3. microRNA Modulating Therapeutics

MicroRNAs (miRNAs), short non-coding RNAs of approximately 22 nucleotides, play a crucial role as post-transcriptional regulators, influencing gene expression by either inhibiting translation or promoting mRNA degradation [154]. Their targeting mechanism involves complementarities between miRNA positions 2–8 and the 3‘-untranslated region of target mRNAs [155]. While a single miRNA can regulate multiple genes, inducing significant changes in the transcriptional landscape, these molecules are highly conserved across mammalian species. Such characteristics make miRNAs attractive therapeutic targets for various diseases, including CVD [156]. Modulating miRNA levels is achieved through synthetic double-stranded miRNAs, viral vector-based overexpression, or chemically modified anti-miR oligonucleotides [156]. Experimental studies on hindlimb ischemia models demonstrate that specific miRNAs, such as *miR-92a*, *miR-21*, and *miR-33*, can profoundly impact transcriptional networks related to angiogenic responses, myocyte growth, fibrosis, and hypertrophy [157].

MiRagen Therapeutics is a clinical-stage biopharmaceutical company, dedicated to advancing miRNA-based therapeutics in CVD [158]. A phase 1 clinical trial (NCT03603431) investigated the local intradermal injection of MRG-110, an oligonucleotide inhibitor targeting *miR-92a* [159]. This study assesses its impact on angiogenic response and wound healing in healthy volunteers with excisional wounds. The potential pro-angiogenic effects of *miR-92a* inhibition lay the groundwork for forthcoming phase 2 clinical trials exploring MRG-110’s therapeutic efficacy in patients with ischemic cardiomyopathy, heart failure, and/or peripheral artery disease [160].

Regulus Therapeutics, in parallel, is actively engaged in the development of miRNA therapeutics for CVD. Their focus includes anti-miR-21, anti-miR-155, and anti-miR-33, targeting fibrotic diseases, inflammation, and cardiometabolic disorders, respectively [161,162,163]. For instance, targeting *miR-33a* with ASOs in atherosclerosis models has proven atheroprotective, influencing cholesterol efflux and plaque size. However, challenges such as systemic delivery-induced effects in non-target tissues necessitate innovative solutions [162].

The context of myocardial infarction unveils the critical role of miRNAs such as *miR-1*, *miR-92a*, and *miR-21*. Their dysregulation influences cardiac function, arrhythmias, and hypertrophy [164]. Interventions using anti-miR-1 antagomirs, liposomes coated with anti-cardiac troponin I antibodies, and engineered dendrimers exhibit potential in mitigating adverse effects post-MI [165,166,167]. Moreover, *miR-92a* inhibition through various delivery systems like polymer NPs and gelatin hydrogel microsphere sheets showcases enhanced angiogenesis and cardiomyogenesis, indicating a multifaceted approach to myocardial infarction treatment [168]. Extracellular vesicles (EVs) loaded with anti-miR-21 present a novel avenue, demonstrating reduced fibrosis and improved cardiac function [169].

In a breakthrough study, *miR-132* gene therapy proves instrumental in controlling cardiac hypertrophy, particularly in a novel porcine model of pressure-overload-induced heart failure. Targeting PPARGC1A/NFE2 signaling, focusing on *SIRT1*, anti-miR-132 emerges as a potential therapeutic strategy for heart failure patients, showcasing its pivotal role in mediating pathologic heart hypertrophy [170,171]. *MiR-132-3p* (*miR-132*) exhibits elevated cardiac expression under cardiomyocyte stress, contributing to progressive cardiac remodeling and heart failure events [172]. This synthetic oligonucleotide *CDR132L*, designed to inhibit *miR-132*, demonstrated groundbreaking potential in pre-clinical studies by significantly improving cardiac function and even reversing heart failure. Täubel et al.’s phase 1b clinical trial confirms *CDR132L’s* safety and tolerability in humans, showcasing its positive impact on heart failure biomarkers [159]. These studies underscore the potential of miRNA-targeted therapies across various cardiovascular conditions, offering insights into innovative delivery methods and therapeutic outcomes.

### 4.4. Modified mRNA Therapeutics

mRNA, with its direct translation into proteins, holds a dual significance in therapy: firstly, by rectifying gene expression deficiencies through exogenous mRNA introduction, and secondly, by integrating mRNA into vaccines. Notably, mRNA therapies for CVDs have explored [173]. Zangi et al. utilized chemically modified mRNA (*modRNA*) that encodes for vascular endothelial growth factor-A (*VEGF-A*) for the treatment of myocardial infarction in mice [174]. Another study examined the impact of *VEGF-A modRNA* in individuals with type 2 diabetes mellitus, showing elevated VEGF-A protein levels and increased skin blood flow in the treated group compared to the control (saline) group [175]. Clinical trials, such as the randomized phase 1 study (NCT02935712), introduced *VEGFA* mRNA in healthy volunteers with type 2 diabetes mellitus. This study demonstrated the induction of *VEGF-A* production without serious side effects. Skin microdialysis revealed increased local *VEGF-A* levels, peaking at 3.5–5 h and sustained for 24 h, supporting the safety and tolerability of *VEGFA* mRNA[175]. In a phase 2a safety and exploratory efficacy study (EPICCURE) in patients with reduced left ventricular ejection fraction (LVEF) undergoing coronary artery bypass grafting, 30 epicardial injections of *VEGFA* mRNA were administered [176]. Despite recruitment challenges and the study’s termination after 11 patients, preliminary results indicated meeting the primary endpoint of safety and tolerability, supporting further clinical development.

## 5. Recent Advancements in Gene Editing Therapeutics

The advancement in gene editing has opened up new possibilities for addressing genetic disorders, particularly CVD [177]. Five primary techniques for gene editing were established, namely CRISPR, TALENs, ZFNs, base editing (BE), and RNA editing [178]. These methods facilitate precise and personalized therapies for a variety of genetic disorders. CRISPR is a groundbreaking gene-editing tool known for its exceptional precision. This technology operates in conjunction with the Cas9 enzyme, which acts as molecular scissors to precisely cut DNA at specific target sites. This synergy enables accurate and efficient modifications to the genome, establishing CRISPR-Cas9 as a highly powerful tool in the field of gene editing [179]. Focusing on CVD, researchers are exploring its potential to correct genetic mutations associated with conditions like familial hypercholesterolemia [180,181]. By precisely targeting and modifying specific genes related to lipid metabolism or cardiovascular function, CRISPR holds the potential to pave the way for tailored therapeutic interventions that address the root causes of cardiovascular disorders [182]. The advent of BE adds a novel dimension to gene editing, allowing for the direct conversion of one DNA base pair into another without causing double-strand breaks [105]. In the realm of CVD, BE holds promise for correcting point mutations linked to conditions such as hypertrophic cardiomyopathy. The ability to precisely alter specific nucleotides provides a unique advantage in addressing the underlying genetic causes of CVD [105].

TALENs and ZFNs offer precision in targeting genes associated with CVD. TALENs empower researchers to engineer proteins with specific DNA-binding capabilities, allowing for the modification of genetic factors contributing to the development and progression of CVDs, such as atherosclerosis or cardiac hypertrophy [183]. Similarly, Zinc Finger Nucleases (ZFNs) provide a customizable approach, enabling targeted modifications in the genetic code related to dyslipidemia or cardiac arrhythmias [180,184]. RNA editing adds another dimension, influencing gene expression in CVD by modifying the nucleotide sequence of RNA molecules [185,186]. These dynamic tools offer a subtle strategy for fine-tuning gene expression, addressing the complex landscape of CVD with potential therapeutic interventions.

In this section, the latest advancements and ongoing clinical trials in the fields of gene editing for CVD will be discussed, with a focus on providing a concentrated exploration of the cutting-edge developments that are shaping these transformative areas of medical research.

### 5.1. Editing PCSK9 and ANGPTL3 Genes

A study in a mouse model utilized CRISPR/Cas9 to target the *PCSK9* gene, resulting in a 50% mutation rate, significantly lowering the concentration of PCSK9 and cholesterol (30–40%) in plasma, and increasing the expression of LDLR on the liver, without any off-target events in the specified loci [187]. Correction of the H530R mutation in the *PRKAG2* gene using CRISPR/Cas9 restored heart morphology and cardiac function in mice with cardiac hypertrophy [188]. Verve Therapeutics is advancing a liver-directed therapy for familial hypercholesterolemia by disrupting the *PCSK9* gene using a base editor, mitigating undesirable on-target effects caused by double-strand breaks (DSB) [189]. They employ LNPs for transiently delivering the base editor as mRNA. Adenine base editors (ABE) testing in macaques, utilizing a guide RNA (gRNA) with a human-identical target, demonstrated sustained *PCSK9* knockdown, reduced LDL-C levels, and minimal off-target effects even 8 months post-injection [189]. Acuitas Therapeutics also achieved successful *PCSK9* knockdown in macaques with a single LNP dose of ABE [190]. While initially targeting heterozygous familial hypercholesterolemia, this technology holds broader potential for addressing common hyperlipidemias, thereby reducing the CVD risk.

BE, targeting *ANGPTL3*, demonstrated reductions in TGs in both wild-type (31%) and HoFH mice (56%). Deep sequencing in the BE-*ANGPTL3* group revealed a 35% editing rate, showcasing the precision of the editing process with no observed off-target effects [191].

### 5.2. Editing ApoC-III Gene

Past research has established a positive correlation between *ApoC-III* and CVDs [192]. Guo et al. utilized CRISPR/Cas9 to knock out the *ApoC-III* gene in Syrian golden hamsters, whose lipid metabolism mirrors that of humans. The *ApoC-III* (−/−) hamsters exhibited lower TG levels, and those on a high-cholesterol, high-fat diet displayed reduced atherosclerosis in thoracic and abdominal arteries, aligning with *ApoC-III*-deficient patient profiles [193]. Another study employed CRISPR/Cas9 to establish an *ApoC-III* (−/−) rabbit model, revealing a 50% reduction in TG levels under a normal diet, along with increased plasma LPL. When subjected to a high-fat diet, *ApoC-III* knockout demonstrated enhanced capability in maintaining low levels of plasma TG, total cholesterol, and LDL-C [194].

### 5.3. Editing TTR Gene

A study by Finn et al. showed a significant reduction of 97% in TTR protein levels in mouse plasma after treatment with CRISPR/Cas9 targeting the *TTR* gene. In a groundbreaking move, Intellia initiated the first CRISPR/Cas9 liver-directed gene editing for treating *ATTR* (NCT04601051). This therapy employs LNP delivery of chemically modified mRNA encoding SpCas9 and gRNA targeting *TTR* [195]. Indels in the *TTR* gene prevent the production of toxic misfolded proteins by the liver. At 4 weeks post-injection, patients receiving the higher dose experienced an 87% reduction in *TTR*, with no off-target editing and only mild adverse effects [196]. These highly promising results underscore the tremendous potential of CRISPR/Cas9 technology in treating and preventing human diseases.

### 5.4. Challenges in the Development of Gene-Editing Pharmaceuticals

The rapid advancement in gene-editing technologies, notably CRISPR-Cas9, has revolutionized our understanding of biology and disease mechanisms, offering unprecedented possibilities in human genetics and genetic disorder treatments [109,197]. Despite its advantages—simplicity, precision, high efficiency, and simultaneous multi-site editing—CRISPR/Cas9 faces challenges hindering broad clinical use.

Foremost among these challenges is the intricate structure of the CRISPR/Cas9 system, coupled with an incomplete understanding of its clinical implications. Questions persist about the existence of a naturally occurring protein more precise and efficient than Cas9 [198]. Moreover, the precise DNA-targeting mechanism of CRISPR/Cas9, while generally accurate, may exhibit off-target effects, cutting DNA sequences differing by a few bases from the target sequence [199]. Despite ongoing efforts to minimize off-target occurrences, the intricacies and gaps in genetic knowledge pose persistent challenges.

Another concern revolves around the use of bacterial-produced Cas9 protein, potentially triggering detrimental immune responses in hosts [200]. The extent to which the self-destruction mechanism of the protein can mitigate this risk remains uncertain. Additionally, the expression of Cas9 may activate the p53 pathway, raising further safety considerations [185]. The requirement for a protospacer adjacent motif (PAM) near the target site limits CRISPR/Cas9’s applicability, introducing a constraint that needs addressing [201]. Despite these challenges, recent advancements in gene-editing techniques, such as base editors and prime editing, enhanced safety. Cytosine and adenine base editors provide precise mutations, while prime editing allows complex modifications without double-strand breaks [202]. Innovations such as anti-CRISPR proteins and modified gRNA contribute to regulation. However, the permanence of DNA changes demands cautious safety assessments, addressing unintended disruptions and off-target effects. Ethical debates underscore the need for limited clinical use and ongoing research to refine safety profiles [203].

## 6. Recent Advancements in Immunotherapy

The emergence of immune checkpoint inhibitors (ICIs) like PD-1, PD-L1, and CTLA-4 has demonstrably revolutionized cancer treatment by leveraging the immune system’s inherent anti-tumorigenic potential [204]. However, recent studies exploring the potential of CTLA-4 blockade in atherosclerosis have yielded unexpected results. Poels et al. demonstrated in Ldlr−/− mice, fed a 0.15% cholesterol diet for six weeks, a two-fold increase in atherosclerotic lesions in the aortic arch after CTLA-4 antibody treatment compared to an isotype control. Interestingly, T-cell activation was induced without altering monocyte/macrophage content in the plaque [205]. Similar findings were observed with a combination of CTLA-4 and PD-1 antibodies in hyperlipidemic mice [206]. The research for using ICIs as therapy for atherosclerosis is still in its early stages, and several significant challenges need to be addressed before they can be safely and effectively used for this disease.

Emerging research is exploring exciting vaccination strategies as a potential tool to prevent and even reverse atherosclerosis by stimulating the immune system to regulate LDL-C levels. One promising target is PCSK9 (proprotein convertase subtilisin/kexin type 9), a liver protein that plays a key role in LDL-C regulation [207]. A recent study by Madeline et al. evaluated VXX-401, a peptide-based vaccine against PCSK9 in non-human primates. Their results showed a significant reduction in the LDL-C levels by one-third [208], prompting them to initiate a phase 1 clinical trial (NCT05762276) involving 12 participants. It is important to note that other approaches targeting PCSK9, such as siRNA [209] and mAbs [210], are also being investigated.

Another potential immunotherapeutic approach for atherosclerosis involves targeting pro-inflammatory cytokines to modulate their disease-promoting role. mAbs against TNF-α, IL-1β, and IL-6 were extensively investigated in vitro, in vivo, and in clinical trials to assess their efficacy [211,212,213]. As mentioned before, canakinumab, an anti-IL-1β mAb, was evaluated in the CANTOS trial (NCT01327846), with an average follow-up of 3.7 years. The results demonstrated a significant reduction in recurrent cardiovascular events (e.g., heart attacks and strokes) compared to a placebo [44]. Despite these promising findings, canakinumab is not currently approved for atherosclerosis treatment due to the increased risk of fatal infections observed in the trial [214].

Chemokines, key mediators of immune and inflammatory responses through cell recruitment, activation, development, and maturation, have emerged as promising targets for preventing and treating diseases [215]. Strategies, such as suppressing chemokine–receptor interactions using antagonists or modulating receptor expression, represent potential immunotherapeutic approaches for atherosclerosis [216]. Table 1 provides examples of chemokines and their corresponding receptors that were investigated as potential therapeutic targets for atherosclerosis at both pre-clinical and clinical stages.

The imbalanced gut microbiota has recently gained attention for its role in disease development, including CVDs [221]. In atherosclerosis, numerous studies suggest that certain gut bacteria may influence inflammation and plaque formation [222,223,224,225]. Manipulating the gut microbiota through probiotics or prebiotics could offer preventive or therapeutic benefits for immune system balance and controlling cholesterol levels. Jurairat et al. investigated the effect of up-taking *L. paracasei* TISTR 2593 as a supplement for 90 days, improving the inhibition of the pro-inflammatory cytokines, mainly TNF-α, and lowering the level of LDL-C [226]. On the other hand, improving the microbiota activity status and enhancing the anti-inflammatory benefits could be achieved indirectly through prebiotic molecules such as inulin [227].

## 7. Nanotechnology for Targeted Therapy of Atherosclerosis

In recent decades, significant progression in the treatment approaches for atherosclerosis has been acknowledged due to the involvement of nanotechnology in the development of new therapeutic agents. The emergence of nanotechnology in the management of atherosclerosis disease is a promising approach to enhance the impact of the current atherosclerosis therapy and also to develop new novel therapeutic agents [228]. There are two potential approaches for carrying NPs through atherosclerosis, one is by enhanced permeability and retention (EPR) mechanism and the latter is by active targeting of overexpressed receptors [229]. As described in the previous sections, one of the main pathophysiological features of atherosclerotic plaques is vessel dysfunction and endothelium injury. The exploiting of the nanotechnology approach to promote the penetration of loaded therapeutic materials to the targeted diseased tissue was considered, which resulted in the accumulation of therapeutic materials in targeted cells, and consequently, improved the treatment efficiency [230].

There is an advanced understanding of CVD pathogenesis, but there is a lack of nanomedicine research compared to other life-threatening diseases like cancers. The management of atherosclerosis disease is mainly based on using steroidal anti-inflammatory drugs such as corticosteroids. The formulation of a popular drug therapy regimen, a nanosized dosage form, was considered an effective promising therapeutic approach that could be applied in clinics [231]. It was demonstrated that the administration of LNPs encapsulated prednisolone as a corticosteroid drug and enhanced its accumulation in plaque macrophages, hence, promoting the disruption of atherosclerotic macrophage [232]. Moreover, Valk et al. reported that the in vitro application of prednisolone fabricated as LNPs could induce macrophage toxicity, thus improving the therapeutic effect of anti-inflammatory drugs in atherosclerosis management [233].

In addition to the corticosteroid anti-inflammatory medications, the anti-proliferative drugs were also highlighted as one of the therapeutic approaches in atherosclerosis management and used in therapeutic regimens [234]. Increasing the area of necrosis in atherosclerosis tissue was recognized following the analysis of in vivo samples indicating the presence of a hypoxic microenvironment in the plaques. The formulation of sirolimus and paclitaxel as NPs exhibited significantly higher anti-proliferative effects, especially under hypoxia conditions in comparison to the free drugs [235]. These results suggested the importance of nanotechnology in the improvement of currently applied approaches in atherosclerosis management. A summary of different nanosized therapeutic approaches that can be used in the treatment of atherosclerosis is shown in Table 2.

### 7.1. Strategies for Active Targeting of Atherosclerosis

#### 7.1.1. Active Targeting of Macrophages

Immune cells such as monocytes and macrophages play a key function in atherosclerosis disease progression. Due to the ability of macrophages to travel into the inflammatory tissue, these phagocytic cells are considered as one of the main imaging and therapeutic candidates for the targeting of atherosclerosis plaques [247]. A study on the active targeting of macrophages in inflammatory atherosclerotic plaques in ApoE knockout mice using iron oxide NPs decorated with the mAb (anti-CD163) demonstrated high active targeting with a dose of 36 μmol Fe per kg, whereas the non-targeted iron oxide NPs needed a much higher dose of 500–1000 μmol Fe per kg [236,248]. The active targeting of overexpressed CD44 receptor in macrophage using iron oxide conjugated with hyaluronic acid (HA) polysaccharide was applied in an atherosclerotic rabbit model, while unconjugated NPs required around a 10 times higher amount of Fe per kg to be able to bind the CD44 receptor onto the surface of targeted macrophage [237].

In addition to the previously mentioned approaches, the active targeting of macrophages can be performed by coating NPs with oxidized phosphatidylcholine lipid that has a high natural affinity to bind overexpressed CD36 receptors on the surface of macrophages [238]. Targeting the cytokines expressed by foamy macrophages, i.e., osteopontin, using iron oxide NPs decorated with anti-osteopontin mAb could enhance the targeting of atherosclerosis plaques [231]. Annexin V molecule was also used to target apoptotic macrophages in atherosclerotic plaques by coating nanocarriers with this protein, and therefore, attaching to the phosphatidylserine present on the membrane of apoptotic macrophages [249]. Peptides can also be implemented in the targeting of macrophages. For instance, Hirulog peptide inserted into engineered Simian virus 40 (SV40) NPs was reported to efficiently target macrophages [239].

The active targeting of macrophages could be promoted significantly by adjusting the physicochemical parameters of the synthesized NPs through the addition of sugar-based amphiphilic macromolecule (AMs) shells [250]. The resulting NPs will have structural and physicochemical properties to mimic the oxidized lipoproteins, and this construct might enhance the binding of modified NPs to the scavenger receptors of macrophages such as CD36 and macrophage scavenger receptor 1 (MSR1) [251].

#### 7.1.2. Active Targeting through Adhesion Molecules

The endothelial cells in atherosclerotic plaques usually overexpressed several adhesion molecules such as selectins, intracellular adhesion molecule-1 (ICAM-1), and vascular cell adhesion molecule-1 (VCAM-1), also referred to as CD106 [252]. These adhesion molecules could be targeted using NPs decorated with some short peptides to increase the therapeutic efficiency in atherosclerosis. For instance, a selectin adhesion molecule was reported to target via fabricated particles encapsulated with microRNA (*miR-146a/-181b*) as an athero-protective agent [240]. The delivery of PLGA NPs loaded with anti-ICAM-1 to macrophage plaque is a targeting approach via the cell adhesion molecule-mediated endocytosis pathway of endothelial cells [231]. Furthermore, VHPKQHRAEEAK is a short peptide that is demonstrated to target VCAM-1 in atherosclerotic plaques in vivo using magnetic NPs [241]. Five different endothelial cell adhesion molecules were knocked down and downregulated in ApoE double knockout mice using targeted siRNA-loaded into polymeric nanocarriers and results showed significant monocyte recruitment inhibition of atherosclerotic lesions [249].

#### 7.1.3. Active Targeting of Extracellular Matrix

In the case of atherosclerosis progression, the bases of blood vessels, especially collagen IV, were demonstrated to be exposed [253]. Hence, collagen IV is considered one of the main ECM targets in atherosclerosis. Collagen IV binding peptide (Col IV-tg-) was formulated as iron oxide NPs and exhibited to perform the successful targeting of atherosclerosis [254]. Another ECM molecule that is overexpressed in atherosclerosis and could be targeted is the actin-binding protein (profilin-1) [255]. The in vivo knockdown of this upregulated protein using profilin-1 siRNA-loaded into inorganic NPs (i.e., gold NPs) was demonstrated to reduce the proliferation and migration of smooth muscle cells and improve the therapeutic efficiency in the atherosclerosis model [242]. The PLGA and PEG polymeric NP payload with IL-10 were developed to inhibit the inflammatory cell accumulation and facilitate their clearance. The prepared NPs penetrated the injured endothelial tissue through the EPR mechanism and interacted with the overexpressed collagen IV, and data revealed that a reduced formation of atherosclerotic plaques was achieved [243].

#### 7.1.4. High-Density Lipoprotein Nanoparticles for Atherosclerotic Plaques Targeting

The human endogenous HDL was reported to bind lipid-laden plaque macrophages, and this approach is implemented in the atherosclerotic plaques targeted by fabrication of HDL mimetic NPs [256]. PLGA-HDL NPs conjugated with ApoA-1 and phosphocholine and loaded with hydrophobic drugs such as statins showed a controlled release effect [244]. One of the important advantages of using polymeric NPs (i.e., PLGA) is the spherical shape of resulted particles mimicking the native HDL. The application of formulated polymeric NPs in ApoE knockout mice demonstrated a high accumulation of administrated NPs in macrophages residing in atherosclerotic plaques [244].

#### 7.1.5. Hyaluronic Acid (HA) Based Nanoparticles for Atherosclerotic Plaques Targeting

One of the common characteristic features of atherosclerotic plaques is the overexpression of CD44 glycoprotein. Naturally, HA has a high affinity to bind CD44 receptors, and this strategy can be used to facilitate drug delivery to targeted tissue [257]. It was reported that PLGA NPs loaded with simvastatin and coated with HA and administrated in vivo by atherosclerotic rabbit models indicated better low hepatic clearance and improved athero-protective activity [231]. Moreover, the accumulation of HA-coated NPs in the targeted atherosclerotic plaques was improved, owing to the interaction of conjugated polysaccharides with overexpressed CD44 receptors [245]. The therapeutic efficiency of atorvastatin-loaded polymeric NPs in atherosclerotic plaque mice was improved significantly by the conjugation of HA on the surface of these NPs [245].

#### 7.1.6. Solid Lipid Nanoparticles for Atherosclerotic Plaques Targeting

One of the therapeutic approaches in the management of chronic inflammatory diseases like atherosclerosis is using immune modulators, such as methotrexate, which is reported to control the released proinflammatory cytokines [258]. The preparation of methotrexate as solid–lipid NPs (SLNs) was demonstrated to reduce the production of TNFα and IL-6 proinflammatory cytokines significantly following the incubation with J774.A1 macrophages compared to the free drug [231]. It was demonstrated that the injection of amiodarone-loaded LNPs as an immune modulator enhanced the therapeutic activity and reduced the toxicity in rat models [246].

It should be noted that the understanding of the protein corona, a layer surrounding the NP, is crucial for designing a safe and efficient atherosclerosis treatment. The corona’s composition can affect the NP interaction and drug-release profiles; therefore, it could interfere with the therapeutic efficacy [259]. New strategies like zwitterionic coatings and controlling corona structure can reduce protein corona’s obstructive effect in drug targeting and release [260]. Moreover, the conjugation of PEG on the outer surface of designed NPs is an additional strategy that is reported to reduce the effect of protein corona adhesion [261].

## 8. Recent Advancements in Diagnosis

### 8.1. Diagnostic Application of Targeted Nanomedicine

Recent advancements in targeted nanomedicine have significantly contributed to the diagnosis and understanding of atherosclerosis development. Various imaging modalities, including MRI and CTA, were utilized to assess the risk of plaque formation and identify vulnerable plaques at different stages of atherosclerosis. Biomarkers, such as cell adhesion molecules, cytokines, and surface receptors, were targeted for plaque imaging using imaging nanoprobes [262].

In MRI, NP probes like gadofluorine M and ultrasmall superparamagnetic particles of iron oxide (USPIOs) were used as contrast agents to image plaque inflammation. USPIOs are taken up by macrophages in atherosclerotic plaques, leading to a decrease in MRI signal intensity. Clinical studies have shown that USPIO-enhanced MRI can identify plaque inflammatory status and stability. Other specific MRI contrast agents were developed to target various markers and stages of atherosclerosis, including thrombi, plaque neovascularization, macrophages, oxidized LDL, and apoptosis. Modified HDL was used to image macrophages within plaques [262,263].

CCTA is widely used in clinical settings due to its short acquisition times, but it is less sensitive for contrast visualization compared to MRI. NPs have been explored as X-ray contrast agents for CT imaging. For example, iodinated NPs were used to detect macrophages in atherosclerotic plaques, resulting in signal enhancement. Multicolor CT techniques using photon-counting detectors can distinguish among different contrast agents and endogenous structures, enabling the identification of macrophage-rich lesions and thrombi [262,264]. Other imaging techniques such as PET and SPECT, i.e., single-photon emission computed tomography, offer high sensitivity for detecting molecular targets in atherosclerosis but lack anatomical information. They are often combined with CT or MRI for better visualization. Various radioactive tracers and NPs have been developed for nuclear imaging to quantify arterial inflammation and detect different aspects of atherosclerotic disease [239,249,262,263,264].

Optical imaging techniques such as fluorescence molecular tomography, photo-acoustic imaging, fluorescence, absorption and Raman spectroscopy, optical coherence tomography, and bioluminescence are being explored for molecular imaging of atherosclerosis. However, optical and ultrasound techniques face limitations in imaging deep tissues, and invasive catheter-based imaging systems have been developed for imaging centrally located blood vessels. Ultrasound-mediated imaging favors the use of larger probes like microbubbles and larger liposomes [249,262,263,264]. For instance, VCAM-1, an adhesion molecule expressed on activated endothelial cells, was used as a biomarker for abnormal endothelial cell targeting. mAbs that target cell adhesion molecules, including ICAM-1, VCAM-1, and P-selectin, were conjugated to imaging nanoprobes to detect vascular inflammation and atherosclerosis. In early-stage inflammation, monocytes attach to the endothelium through adhesion molecules and migrate into the vascular intima. This process involves molecules like MCP-1, GM-CSF, and CXCL4, which can be used as imaging targets [239].

Macrophages play a crucial role in atherosclerosis, and their imaging has been facilitated by targeting molecules such as MARCO, SR-AI, CD68 receptors, CD44 receptors, IL-6 cytokines, and osteopontin (OPN). SR-A, an overexpressed surface receptor on foamy macrophages, was used as a biomarker for molecular imaging of atherosclerotic plaques. HA is another targeting ligand that can specifically bind to the macrophage surface receptor CD44, which is involved in foamy macrophage formation. Inflammatory biomarkers like MPO, secreted by macrophages in advanced-stage atherosclerosis, can serve as active targets for detecting vulnerable plaques and stratifying the stages of the disease [249].

Profilin-1, an intracellular actin-binding protein overexpressed in atherosclerotic plaques, has shown potential as a target for diagnosis and therapy. Furthermore, neovascularization, induced by hypoxia and angiogenic factors like *VEGF*, plays a role in plaque development. Targeting neovascularization can enhance the understanding of plaque progression and promote the detection of LDL infiltration through leaky micro-vessels [239].

In summary, recent advancements in targeted nanomedicine have facilitated the diagnosis and treatment of atherosclerosis by targeting various biomarkers and processes involved in plaque development. These innovative approaches provide valuable insights into plaque characterization, disease progression, and potential therapeutic strategies.

### 8.2. Diagnostic Application of Nanotheranostics

Recent advancements in the diagnostic application of a nanotheranostics approach for atherosclerosis and CVD have shown remarkable innovation in disease management. Nanotheranostics, which combine therapeutic and diagnostic functionalities within a single nanoscale platform, offer unprecedented opportunities for precise and personalized treatment strategies. One notable advancement is the development of multifunctional NPs that can simultaneously deliver therapeutic agents, such as anti-inflammatory drugs or gene therapies, while also incorporating imaging probes for real-time monitoring of the treatment response and disease progression^1^. Another approach involved the development of MRI-based theranostic NPs, such as HDL-MNS, which mimic natural HDL and show potential as a contrast agent for MRI. SLNs loaded with prostacyclin and USPIOs were used for image-guided therapy, inhibiting platelet aggregation and providing increased MRI signal [239].

Other NPs, such as cerium oxide-coated iron oxide NPs, chitosan nano-cocktails, and paramagnetic perfluorocarbon NPs, have shown promising results in reducing the reactive oxygen species (ROS) and enhancing the MRI contrast. Moreover, targeted nanotheranostic platforms have emerged, utilizing ligand-functionalized NPs to specifically target atherosclerotic plaques and enabling enhanced imaging, accurate diagnosis, and site-specific therapy delivery [242]. The integration of diagnostic and therapeutic capabilities within nanotheranostic systems holds great promise for revolutionizing the management of atherosclerosis and CVD, leading to improved patient outcomes and a more effective approach to disease treatment.

Fluorescence-based theranostic NPs have gained attention for atherosclerosis imaging and treatment. Selenium NPs (SeNPs) coated with folic acid and HA were developed to target inflammatory macrophages, inducing cell death through the generation of toxic singlet oxygen. Dextran sulfate-deoxycholic acid (DS-DOCA) nanomaterials loaded with the photosensitizer chlorin e6 (Ce6) demonstrated high macrophage cell death rates under laser irradiation. Nanogels, such as HASF@Cur, were effective in reducing ROS levels in macrophages. Carbon nanotubes functionalized with Cy5.5 dye were used for near-infrared (NIR) imaging and photothermal ablation of macrophages. Furthermore, synthetic HDL NPs, SV40 NPs, and carboxyfluorescein-PEG micelles have shown potential for the diagnosis and treatment of atherosclerosis through their excellent targeting, biocompatibility, and therapeutic properties. However, the use of fluorescence agents in clinical diagnosis may be limited due to high background fluorescence in the human body [239,242,264].

Gold NPs were explored as CT contrast agents and photo-thermal agents for atherosclerosis theranostics. Copper sulphide NPs (CuSNPs) were investigated for photoacoustic imaging, showing a reduction in lipid accumulation and foam cell formation upon NIR laser irradiation. Multiagent-based theranostic NPs, including magnetic fluorescent NPs and lipid-latex (LiLa) NPs, have demonstrated a specific aggregation in areas with inflammatory macrophages and foam cells, leading to a reduction in plaque areas. These NPs have potential applications in MRI and fluorescence imaging [242].

Overall, nanotheranostic approaches offer promising prospects for the diagnosis and treatment of atherosclerosis. Iron oxide-based MRI is considered a promising diagnostic strategy, while targeted NPs and drug-loaded nanosystems showed improved treatment effects and imaging capabilities. Future research should focus on optimizing the specificity and efficacy of targeted theranostic NPs for atherosclerosis.

## 9. Ongoing Clinical Trial on Atherosclerosis Diagnosis and Treatment

A groundbreaking clinical trial is currently underway to revolutionize the diagnosis of atherosclerosis. Researchers are harnessing the power of biomarkers and advanced imaging techniques to identify unstable atherosclerotic lesions with unprecedented accuracy. By analyzing specific microRNAs associated with these lesions, the trial aims to unravel their intricate relationship with disease progression. Additionally, the trial explores the impact of plasma trimethylamine N-oxide levels on the advancement of atherosclerotic plaques, shedding light on a previously unexplored avenue. Another innovative trial focused on developing a state-of-the-art deep learning model that can automatically detect carotid plaques, assess the lumen stenosis rate, and evaluate plaque stability using dynamic ultrasound and contrast-enhanced ultrasound images. This groundbreaking approach has the potential to enhance the efficiency and accuracy of carotid plaque assessment, particularly in underserved regions lacking access to experienced sonographers. These remarkable advancements, coupled with studies investigating plaque stiffness and the correlation between stress testing and plaque composition, are poised to transform the field of atherosclerosis diagnostics. With these ongoing clinical trials pushing the boundaries of medical knowledge, a new era of precision medicine for atherosclerosis is on the horizon [265]. Details of the recent ongoing trials follow.

ClinicalTrials.gov ID NCT05680935 is an ongoing clinical trial at I.M. Sechenov First Moscow State Medical University. The study aims to identify biomarkers of unstable atherosclerosis in brachiocephalic arteries. It focuses on specific microRNAs associated with unstable atherosclerotic lesions and their relationship with lesion progression. Additionally, the trial investigates the impact of plasma trimethylamine N-oxide levels on the progression of atherosclerotic lesions.

ClinicalTrials.gov ID NCT05230576, sponsored by Jia Liu, is another ongoing trial. It aims to develop a deep learning model for the automatic and accurate detection of carotid plaques, calculation of lumen stenosis rate, and evaluation of plaque stability using dynamic ultrasound and contrast-enhanced ultrasound images. The goal is to provide a comprehensive assessment of cardiovascular risk associated with carotid plaques, particularly in areas with limited access to experienced sonographers.

ClinicalTrials.gov ID NCT06214429 is a prospective diagnostic accuracy cohort study sponsored by UPECLIN HC FM Botucatu Unesp. The trial aims to compare the accuracy of carotid atherosclerotic plaque stiffness assessed by shear wave elastography (SWE) with greyscale median values (GSM), MRI, and histopathological findings. The study evaluates the correlation between plaque stiffness and GSM values, MRI findings, and histopathological findings in participants with carotid plaques causing stenosis above 50%.

ClinicalTrials.gov ID NCT05416385, sponsored by Dr. Amer Johri at Queen’s University, aims to improve the accuracy of stress tests in diagnosing heart disease. The study combines stress testing with neck ultrasound to assess plaque composition. By identifying patients with leaky plaques using ultrasound, the trial aims to better identify individuals at risk for cardiovascular events. The study will follow patients from multiple sites in Canada for three years to evaluate the cardiac outcomes.

ClinicalTrials.gov ID NCT04758650 is a phase 2 study sponsored by Universitair Ziekenhuis Brussel. The trial evaluates the clinical potential of 68GaNOTA-anti-MMR-VHH2, a PET imaging agent, in various conditions, including atherosclerosis. The objective is to assess in vivo imaging of MMR-expressing macrophages using PET, providing insights into the presence and distribution of these macrophages in different diseases.

These ongoing trials represent important research efforts aimed at advancing our understanding of atherosclerosis, improving diagnostic accuracy, and ultimately enhancing patient care and outcomes in the field of cardiovascular health.

Recent advancements in ongoing clinical trials for the therapeutic approach to atherosclerosis have shown promising results. One study entitled “Rosuvastatin Effect on Atherosclerotic Plaque Metabolism—a Subclinical Atherosclerosis Imaging Study With 18F-NaF PET-CT” aims to evaluate the effect of rosuvastatin on plaque metabolism using 18F-NaF PET-CT imaging. The study focuses on assessing the safety and efficacy of rosuvastatin in improving hemodynamics and facilitating the retention of acute luminal gain in specific arteries.

Another trial entitled “Natural Vascular Scaffolding (NVS) Therapy for the Treatment of Atherosclerotic Lesions in the Superficial Femoral Artery (SFA) and/or Proximal Popliteal Artery (PPA)” is a phase 1 study investigating the safety and preliminary efficacy of NVS therapy in patients with obstructive atherosclerosis. The therapy aims to improve acute hemodynamic outcomes in individuals with lifestyle-limiting claudication.

Another study entitled “Treatment of Patients with Atherosclerotic Disease with Methotrexate-associated to LDL like NPs” explores the use of methotrexate, an anti-inflammatory agent, delivered in cholesterol-rich non-protein NPs. This randomized, double-blind, placebo-controlled study aims to evaluate the safety and efficacy of this treatment in patients with stable coronary disease, specifically assessing its effects on lesion size and inflammation.

One study is investigating the effectiveness of low-dose colchicine in combination with the best medical care in patients with ischemic stroke and ipsilateral atherosclerotic stenosis. The study aims to reduce the risk of major vascular events through colchicine treatment and evaluate the efficacy of ticagrelor compared to aspirin in reducing long-term risks. Another study focuses on evaluating the efficacy and safety of clopidogrel for primary prevention in patients with subclinical coronary atherosclerosis identified on imaging. This trial aims to assess mortality and morbidity rates using MRI imaging. Additionally, a clinical trial is investigating the use of Feraheme for intracranial atherosclerotic disease. The goal is to predict stroke risk and develop improved prevention strategies.

The “Sonodynamic Therapy Manipulates Atherosclerosis Regression Trial on Patients with Carotid Atherosclerotic Plaques (SMART-C)” aims to evaluate the safety and efficacy of sonodynamic therapy (SDT) as a new treatment for carotid atherosclerotic plaque. Another trial, a “Comparative Study of Indobufen and Aspirin in Patients with Coronary Atherosclerosis” aims to compare the antiplatelet efficacy of indobufen and aspirin in patients with coronary atherosclerosis. The “Cysteinyl Leukotriene Antagonist in Atherosclerosis Inhibition in Patients after Endovascular Treatment due to Peripheral Arterial Disease (CADET-PAD)” project investigates the influence of cysteinyl leukotriene receptor antagonists on the reocclusion rate of lower limb arteries in patients with the peripheral arterial disease (PAD) after endovascular treatment.

The “Harmonizing Optimal Strategy for Treatment of Coronary Artery Stenosis—CloPidogREl for Primary preVENTION (HOST-PREVENTION)” trial evaluates the efficacy and safety of clopidogrel for primary prevention in patients diagnosed with coronary atherosclerosis. Another study, the “Randomized Study to Evaluate the Effect of an ‘Inclisiran First’ Implementation Strategy Compared to Usual Care in Patients with Atherosclerotic Cardiovascular Disease and Elevated LDL-C despite Receiving Maximally Tolerated Statin Therapy (VICTORION-INITIATE) (V-INITIATE)” assesses the effectiveness of adding inclisiran to maximally tolerated statin therapy when acceptable LDL-C levels are not achieved.

The “Cardiovascular Outcome Study to Evaluate the Effect of Obicetrapib in Patients with Cardiovascular Disease (PREVAIL)” aims to evaluate the effect of obicetrapib in patients with atherosclerotic cardiovascular disease who are not adequately controlled despite maximally tolerated lipid-lowering therapy. Lastly, the “Effect of Semaglutide in Coronary Atheroma Plaque (SEPLA)” trial evaluates the effect of semaglutide on the burden of coronary atherosclerosis in asymptomatic individuals with type 2 diabetes mellitus who are in optimized and stable treatment with semaglutide.

These ongoing clinical trials aim to advance our understanding of treatment options improve outcomes for patients with atherosclerosis and demonstrate different innovative approaches to atherosclerosis therapy, including the use of statins, vascular scaffolding therapy, and anti-inflammatory agents. These advancements provide hope for more effective, safer, and improved management and prevention approaches for atherosclerosis-related events in the future.

## 10. Conclusions and Future Perspectives

Cardiovascular diseases (CVDs) persist as the leading cause of global mortality, with atherosclerosis playing a central role in the disease pathology. The buildup of lipids and fibrous elements within arterial walls leads to plaque formation and results in chronic inflammation, causing myocardial infarction, ischemic stroke, and peripheral artery disease. Atherosclerotic plaques can evolve into unstable plaques prone to rupture, triggering thrombosis and acute ischemic events. Additionally, hypertension and hypercholesterolemia exacerbate vascular endothelial cell damage, worsening dysfunction and inflammation. Diagnosing atherosclerotic plaques utilizes various imaging modalities, including ultrasound, CT, MRI, and PET, each offering unique advantages and limitations. Despite the effectiveness of current treatments like statins and antiplatelet drugs in reducing CVD risk, emerging therapies aim to further enhance efficacy and safety. Nanotechnology presents a promising avenue in drug delivery, facilitating the treatment of atherosclerotic lesions with novel materials and nanoparticles via active or passive targeting, potentially improving therapeutic outcomes. Furthermore, cell therapy and gene editing represent cutting-edge strategies in cardiac repair and CVD management. BM-MNCs and MSCs are being investigated for their regenerative capabilities. Gene-editing tools such as CRISPR/Cas9 and base editing offer significant potential for correcting genetic mutations associated with CVD. However, challenges remain in ensuring these therapies’ safety, efficacy, and long-term viability. In conclusion, developing innovative strategies, including personalized medicine, advanced imaging techniques, and novel therapeutics, is critical for further reducing the global burden of CVD. Continuous research and clinical trials are essential to translate these scientific advancements into practical clinical applications, ultimately improving patient outcomes in managing cardiovascular diseases.

## Figures and Tables

**Table 1:** A summary of the potential of chemokines and their receptors as target therapy in atherosclerosis.

Chemokine/Receptor Target	Clinical Trials	Outcomes	Further Work	References
CCL2/CCR2	Phase 1, 112 patients (CCR2 antagonism (MLN1202))	Safety outcome with reduction in serum C-reactive protein level	The efficacy of MLN1202 needs to be evaluated in atherosclerosis patients	[217]
CXCL12/CXCR4	No current trials identified	Overexpression of CXCL12 is associated with increased macrophage infiltration in the plaques	Pre-clinical and clinical assessment of the potency and safety of CXCL12/CXCR4 modulation	[218]
CXCL10/CXCR3	No current trials identified	In vivo, administration of the CXCR3 antagonist NBI-74330 demonstrates reduced atherogenesis	Further pre-clinical and clinical evaluations are needed to address concerns about specificity and potential impacts on ant-pathogen immunity	[219]
CCR5	CCR5 antagonist (Maraviroc) on 22 patients	Several markers associated with cardiovascular risk showed improvement compared to a control group	More studies are needed to evaluate the efficacy, address concerns about off-target effects, and investigate potential long-term consequences.	[220]

**Table 2:** Summary of different nanosized therapeutic approaches that can be used in the treatment of atherosclerosis.

Nanoparticles	Loaded Molecule	Target	Reference
Liposome	Prednisolone	Macrophages	[233]
Iron oxide	Anti-CD163 mAb	Macrophages	[236]
Iron oxide-HA	Anti-CD44 mAb	CD44 receptor	[237]
Phosphatidylcholine lipid	Anti-CD36 mAb	CD36 receptor	[238]
Iron oxide	Anti-osteopontin mAb	Osteopontin	[231]
SV40	Hirulog peptide	Macrophages	[239]
Thioaptamer	*miR-146a/-181b*	Selectin	[240]
PLGA	Anti-ICAM-1 peptide	ICAM-1	[231]
Magnetic	Anti-VCAM-1 peptide	VCAM-1	[241]
Gold	Anti-profilin-1 siRNA	Profilin-1	[242]
PLGA-PEG	IL-10	Collagen IV	[243]
PLGA-HDL	Statins	Macrophages	[244]
PLGA-HA	Simvastatin	CD44 receptor	[231]
Polymer-HA	Atorvastatin	CD44 receptor	[245]
Solid lipid	Methotrexate	Proinflammatory cytokines	[231]
Liposome	Amiodarone	Proinflammatory cytokines	[246]

## Data Availability

The authors confirm that the data supporting the findings of this study are available within the article.
